# Relevance of Neutrophil Neprilysin in Heart Failure

**DOI:** 10.3390/cells10112922

**Published:** 2021-10-28

**Authors:** Suriya Prausmüller, Georg Spinka, Henrike Arfsten, Stefanie Stasek, Rene Rettl, Philipp Emanuel Bartko, Georg Goliasch, Guido Strunk, Julia Riebandt, Julia Mascherbauer, Diana Bonderman, Christian Hengstenberg, Martin Hülsmann, Noemi Pavo

**Affiliations:** 1 Department of Internal Medicine II, Division of Cardiology, Medical University of Vienna, 1090 Vienna, Austria; suriya.prausmueller@meduniwien.ac.at (S.P.); georg.spinka@meduniwien.ac.at (G.S.); henrike.arfsten@meduniwien.ac.at (H.A.); stefanie.stasek@meduniwien.ac.at (S.S.); rene.rettl@meduniwien.ac.at (R.R.); philippemanuel.bartko@meduniwien.ac.at (P.E.B.); georg.goliasch@meduniwien.ac.at (G.G.); julia.mascherbauer@meduniwien.ac.at (J.M.); diana.bonderman@meduniwien.ac.at (D.B.); christian.hengstenberg@meduniwien.ac.at (C.H.); noemi.pavo@meduniwien.ac.at (N.P.); 2 Department of Entrepreneurship and Economic Education, Faculty of Business and Economics, Technical University Dortmund, 44227 Dortmund, Germany; guido.strunk@complexity-research.com; 3 Department of Surgery, Division of Cardiac Surgery, Medical University of Vienna, 1090 Vienna, Austria; julia.riebandt@meduniwien.ac.at; 4 Department of Internal Medicine III, Division of Cardiology, Karl Landsteiner University of Health Sciences, University Hospital St. Pölten, 3500 Krems, Austria; 5 Department of Internal Medicine V, Division of Cardiology, Clinic Favoriten, 1100 Vienna, Austria

**Keywords:** heart failure, biomarker, neprilysin, CD10, neutrophils

## Abstract

Significant expression of neprilysin (NEP) is found on neutrophils, which present the transmembrane integer form of the enzyme. This study aimed to investigate the relationship of neutrophil transmembrane neprilysin (mNEP) with disease severity, adverse remodeling, and outcome in HFrEF. In total, 228 HFrEF, 30 HFpEF patients, and 43 controls were enrolled. Neutrophil mNEP was measured by flow-cytometry. NEP activity in plasma and blood cells was determined for a subset of HFrEF patients using mass-spectrometry. Heart failure (HF) was characterized by reduced neutrophil mNEP compared to controls (*p* < 0.01). NEP activity on peripheral blood cells was almost 4-fold higher compared to plasma NEP activity (*p* = 0.031) and correlated with neutrophil mNEP (*p* = 0.006). Lower neutrophil mNEP was associated with increasing disease severity and markers of adverse remodeling. Higher neutrophil mNEP was associated with reduced risk for mortality, total cardiovascular hospitalizations, and the composite endpoint of both (*p* < 0.01 for all). This is the first report describing a significant role of neutrophil mNEP in HFrEF. The biological relevance of neutrophil mNEP and exact effects of angiotensin-converting-enzyme inhibitors (ARNi) at the neutrophil site have to be determined. However, the results may suggest early initiation of ARNi already in less severe HF disease, where effects of NEP inhibition may be more pronounced.

## 1. Introduction

The landmark PARADIGM-HF trial has impressively demonstrated that combined inhibition of the angiotensin II type 1 receptor and neprilysin (NEP) results in significant improved outcomes in patients with chronic heart failure with reduced ejection fraction (HFrEF) [1].

Data from the relatively recent PARAGON-HF study imply that individuals with heart failure (HF) with mildly-to-moderately reduced left ventricular function and distinct subgroups of patients with preserved ejection fraction may also benefit from sacubitril/valsartan therapy [2]. Moreover, emerging evidence suggests that initiating treatment with sacubitril/valsartan early in the course of HFrEF may be beneficial to delay disease progression [3].

In the last few years, cardiovascular (CV) research has been focusing on elucidating the mechanisms of actions related to NEP inhibition (NEPi). However, still, the exact mechanisms involved remain largely unknown.

NEP, also known as cluster of differentiation 10 (CD10), neutral endopeptidase, enkephalinase, or common acute lymphoblastic leukemia antigen, is a zinc-dependent transmembrane metalloendoprotease that is involved in the breakdown of a plethora of vasoactive peptides, including natriuretic peptides, adrenomedullin, angiotensin II, and endothelin-1 [4,5,6]. Predominantly, NEP is found in the plasma membrane of cells in the kidneys, lungs, intestine, brain, and liver and has been shown to be identical to CD10, which is expressed on several distinct hematopoietic cell lineages including neutrophils [4,7].

Regarding the impressive clinical benefits resulting from the inhibition of NEP [1], it may be assumed that the determination of individual enzyme status permits conclusions on treatment response and enables tailored therapy. Considering feasibility, circulating NEP measures are especially compelling in this context. Although NEP is a transmembrane enzyme with a predominantly tissue-based activity, it can be released to the circulation by ectodomain shedding and exocytosis [8,9], while triggers for NEP release are not well elucidated. Numerous studies have described the presence of a non-membrane bound soluble form of NEP (sNEP) in plasma and investigated its prognostic role in several types of HF but also non-HF cohorts [10,11,12,13,14,15,16,17,18]. The data gathered so far revealed conflicting results on the relationship of sNEP with disease states and prognosis, challenging the hypothesis that sNEP is a surrogate for tissue NEP actions and systemic NEP regulation.

In the circulatory system, besides sNEP, the enzyme can be found on neutrophils which express NEP (i.e., CD10) as a surface protein under physiologic conditions. Neutrophil NEP exhibits significant enzyme activity and plays an important role in modulating immune responses [7,19]. Experimental observations have demonstrated that NEP knockout mice display a 10-fold sensitivity to endotoxin stimulation and show higher mortality rates than wildtype animals [20]. A further study identified neutrophil NEP expression as a marker for neutrophil functional capacity with decreased levels found in septic patients [21]. NEP is evidently an enzyme regulating vasoactive state interlinked with inflammatory disposition, both mechanisms fundamentally involved in the progression of HF. Neutrophils provide access to the transmembrane form of the enzyme with functional integrity. The measurement of neutrophil transmembrane neprilysin (mNEP) expression and activity offers an opportunity to gain significant information on circulating and possibly systemic NEP regulation. The role of neutrophil mNEP in HFrEF has been investigated in one pilot study, indeed demonstrating a significant and interestingly inverse association between NEP expression and HF disease state [22].

The aim of this study was to assess neutrophil mNEP expression in a larger cohort of stable HFrEF patients on guideline-directed therapy in order to (i) investigate neutrophil mNEP expression in HF compared to healthy individuals, (ii) correlate neutrophil mNEP with the extent of neurohumoral activation and adverse remodeling, and to (iii) investigate the prognostic role of neutrophil mNEP.

## 2. Materials and Methods

### 2.1. Study Population

Consecutive patients with stable chronic HFrEF on optimal medical therapy, including angiotensin-converting-enzyme inhibitor (ACEi), angiotensin receptor blocker (ARB), as well as angiotensin receptor and neprilysin inhibitor (ARNi) were prospectively enrolled at the Vienna General Hospital, a university-affiliated tertiary care center between 2015 and 2019. Comorbidities, traditional risk factors, routine echocardiographic parameters from a standardized examination, medical therapy, and follow-up data were recorded. Additionally, a cohort of patients with heart failure with preserved ejection fraction (HFpEF) and healthy control subjects were enrolled. The diagnosis of HF with reduced/preserved ejection fraction was defined in accordance to the latest guidelines of the European Society of Cardiology [23]. HFrEF was defined as a history of heart failure with signs and symptoms and a left ventricular ejection fraction below 40%. For the diagnosis of HFpEF the following criteria had to be fulfilled: signs and symptoms of HF, ejection fraction above 50%, and evidence of elevated left ventricular filling pressures. Control subjects were apparently healthy without any previously recorded comorbidities or medications. Exclusion criteria were age below 18 years, active cancer disease, and active infection. Written informed consent was obtained from all participants.

### 2.2. Sampling and Routine Laboratory Analysis

Blood samples were drawn from the cubital vein from all participants at study inclusion. In order to investigate the robustness/timely variance of neutrophil mNEP expression, additional follow-up samples were collected for a subset of HFrEF patients at a subsequent visit within one year of follow-up. Routine laboratory parameters including N-terminal pro-brain natriuretic peptide (NT-proBNP) were analyzed and processed according to the local standards of the Department of Laboratory Medicine of the Medical University of Vienna.

### 2.3. Determination of Neutrophil mNEP Expression by Flow Cytometry

Mean fluorescence intensity (MFI) of CD10 on peripheral neutrophils, i.e., neutrophil mNEP expression, was measured by flow cytometry in whole blood samples. The samples were kept at 4 °C and were analyzed within 4 h after the specimens were obtained. Briefly, 100 μL EDTA-anticoagulated samples were stained with antibodies (CD16 [#335035], CD10 [#332777], BD Biosciences, San Jose, CA, USA). After 30 min incubation in the dark at room temperature red blood cells were lysed using FACS Lysing Solution (BD Biosciences). Thereafter, cells were washed twice with phosphate-buffered saline (PBS) solution and finally the pellet was resuspended in 500 µL of PBS. For all cases, at least 30,000 cells were analyzed in each sample. Neutrophils were gated as FSC^high^SSC^high^CD16^+^ populations. Fluorescence minus one control tubes were used to determine positive–negative thresholds for each antibody during analysis. Flow cytometry was performed with a FACS Canto II system using FACS Diva software 6.1.2 (BD Biosciences).

### 2.4. Determination of NEP Activity in Plasma and the Cellular Compartment Liquid Chromatography-Tandem Mass Spectrometry

In a subset of 40 patients, NEP activity was determined in diluted (PBS, pH 7.4/1 mM ZnCl2) heparinized whole blood and its plasma. After spiking Angiotensin I, samples were incubated at 37 °C in the presence (inhibitor) and absence (solvent) of the NEP inhibitor LBQ-657 (10 µmol/L, Sigma-Aldrich, Saint Louis, MO, USA). Aminopeptidase inhibitor (10 µmol/L, Sigma-Aldrich), Z-Pro-prolinal (10 µmol/L, Sigma-Aldrich), MLN-4760 (10 µmol/L, Sigma-Aldrich), and Lisinopril (10 µmol/L, Sigma-Aldrich) were added to all samples for substrate (Angiotensin I) and product (Angiotensin 1–7) stabilization. Angiotensin quantification of Angiotensin I and Angiotensin 1–7 was conducted as described previously [24]. Specific activity of NEP was calculated by determining the inhibitor sensitive fraction (solvent minus inhibitor) of Angiotensin 1–7 formation. mNEP activity of the cellular compartment was quantified by subtracting the NEP activity measured in plasma from NEP activity in the corresponding whole blood samples.

### 2.5. Echocardiographic Assessment

Standard transthoracic echocardiographic (2D, Doppler) examinations were performed in clinical routine using commercially available equipment (Vivid E7 and E9, General Electric Healthcare, Chicago, IL, USA) according to the current guidelines [25]. Cardiac morphology was assessed in standard four- and two-chamber views. Semi-quantitative assessment of right and left heart function was performed by experienced readers using multiple acoustic windows and graded as normal, mild, mild-to-moderate, moderate, moderate-to-severe, and severe. The grading normal, mild, moderate, or severe refers to a left ventricular ejection fraction of 52–72% for males and 54–74% for females, 41–51% for males and 41–53% for females, 30–40% and <30%, respectively. If present, valvular regurgitation was quantified using an integrated approach and graded as normal, mild, mild-to-moderate, moderate, moderate-to-severe, and severe [26]. Systolic pulmonary artery pressures were calculated by adding the peak tricuspid regurgitation systolic gradient to the estimated central venous pressure.

### 2.6. Follow-Up and Study Endpoints

All-cause mortality was investigated as the primary outcome parameter. Additionally, total CV hospitalization and the combined endpoint of all-cause mortality and total CV hospitalization were assessed. Total CV hospitalization was defined as any planned or unplanned CV event leading to hospitalization.

### 2.7. Statistical Analysis

All statistical analyses were performed using SPSS software (IBM SPSS, Chicago, IL, USA) version 24. Descriptive statistics were expressed as median and interquartile ranges (IQR) for continuous parameters and as percentages and counts for categorical variables. Continuous variables were compared by the Kruskal–Wallis and Mann–Whitney-U-test, counts by the Fisher’s exact test. The correlation between neutrophil mNEP expression and NEP activity was assessed by calculating the Spearman rank correlation coefficient. The paired samples Wilcoxon test was applied to investigate the timely variance of neutrophil mNEP expression. Kaplan–Meier curves using log-rank test were generated to illustrate the association of neutrophil mNEP expression with the endpoints graphically. Cox proportional hazard regression analysis was used to evaluate the effect of neutrophil mNEP expression on outcome measures. Results are presented as hazard ratios (HR) per IQR. To account for potential confounding effects, we formed a clinical confounder cluster encompassing age, kidney function, sex, and NT-proBNP. All tests were two-sided and a *p*-value <  0.05 was considered to be statistically significant.

## 3. Results

### 3.1. Study Population Characteristics

A total of 228 HFrEF patients were included in the study. Baseline demographic, clinical, biochemical, and echocardiographic characteristics are presented in Table 1. Median age of the study cohort was 64 years (IQR: 55–72) and 169 patients (74%) were male. A total of 97 patients (43%) were in New York Heart Association (NYHA) functional class II and 91 (40%) in NYHA III. Approximately half of the study population (n = 127, 56%) patients had a non-ischemic etiology of HF. HF medication was well established with 213 (93%), 217 (95%) and 180 (79%) patients receiving therapy with renin-angiotensin-system blockers, beta-blockers, and mineralocorticoid receptor antagonists, respectively. In total, 101 patients (44%) received ACEi, 50 (22%) ARB, and 62 (27%) ARNi. Additionally, 30 patients with HFpEF and 43 control subjects were enrolled. Table S1 shows the baseline characteristics with respect to the different study cohorts. The median age in the HFpEF population was 76 years (IQR: 71–79), 8 (27%) patients were male. Median age of the control population was 35 years (IQR: 27–51) and 19 (44%) were male.

### 3.2. Heart Failure Is Characterized by Low Neutrophil mNEP Expression

**Figure 1:**
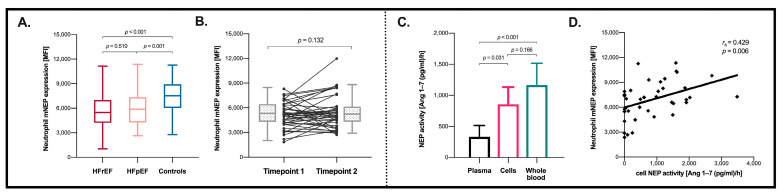
(**A**) Neutrophil membrane-associated neprilysin (mNEP) expression in HFrEF, HFpEF and controls. (**B**) Timely variance of neutrophil mNEP expression. (**C**) Mass spectrometry-based determination of the enzymatic activity of NEP in whole blood, peripheral cells, and plasma (n = 40). (**D**) Scatter plot with linear regression analysis and the Spearman rho correlation coefficient for neutrophil mNEP expression with peripheral cell mNEP activity. Comparison between groups was assessed by using the Kruskal–Wallis test, Mann–Whitney test, and paired Wilcoxon test. Levels of significance are indicated in the respective plots. Tukey boxplots are shown in (**A**,**B**); (**C**) displays geometric means and 95% confidence intervals.

Figure 1A displays neutrophil mNEP expression for the different cohorts. Neutrophil mNEP, expressed as MFI, in HFrEF, HFpEF, and controls was 5474 (IQR: 4211–6998), 5881 (IQR: 4277–7358), and 7520 (IQR: 6029–8940), respectively. Neutrophil mNEP expression was lower in the HF groups as compared to controls (*p* < 0.001 for HFrEF and *p* = 0.001 for HFpEF). There was no difference in neutrophil mNEP expression between the HFrEF and the HFpEF group (*p* = 0.519).

### 3.3. Neutrophil mNEP Is Robust over Time

Short-term follow-up measurements within a time period of 5 months (IQR: 3–8) were available for 42 HFrEF patients on stable HF therapy. Neutrophil NEP expression was individually stable with comparable values for this time interval (*p* = 0.132), as illustrated in Figure 1B.

### 3.4. Blood Cell NEP Activity Is Determined by Neutrophil mNEP Expression

NEP activity of the circulating cellular compartment was markedly increased compared to plasma (666.7 [pg/mL]/h [IQR: 45.6–1510.9] vs. 170.1 [pg/mL]/h [IQR 81.3–345.9], *p* = 0.031) (Figure 1C). NEP has been shown to be almost exclusively expressed by neutrophils [22], correspondingly neutrophil mNEP expression correlated significantly with cell mNEP activity (r_s_ = 0.429, *p* = 0.006) (Figure 1D) but not with sNEP activity (r_s_ = 0.26, *p* = 0.103). Additionally, sNEP activity did not correlate with mNEP activity (r_s_ = 0.160, *p* = 0.323).

### 3.5. Worsening of HFrEF Is Associated with a Continuous Decrease in Neutrophil mNEP

**Figure 2:**
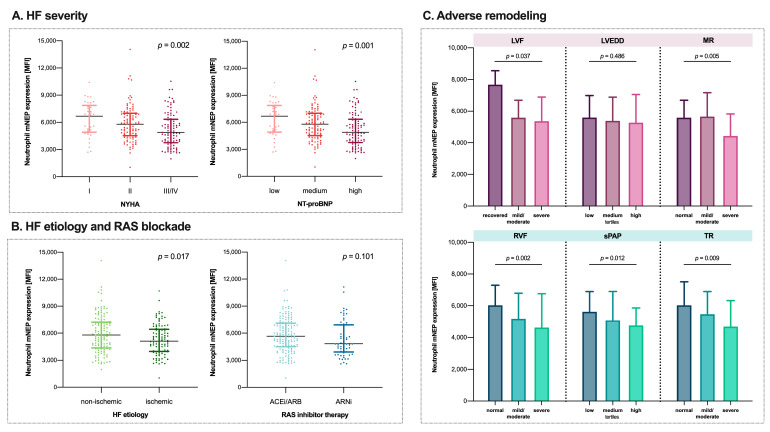
Relationship of neutrophil membrane-associated neprilysin (mNEP) expression with heart failure (HF) severity, reflected by (**A**) New York Heart Association (NYHA) class and tertiles of N-terminal pro-brain natriuretic peptide (NT-proBNP), (**B**) HF etiology (non-ischemic vs. ischemic) and renin-angiotensin-system (RAS) inhibitor therapy, and (**C**) adverse remodeling indicated by echocardiographic indices of left heart function and right heart function. Comparison between groups was assessed by using the Kruskal–Wallis test, levels of significance are indicated in the respective plots. The respective plots display individuals values as well as median and interquartile range. LVF—left ventricular function; LVEDD—left ventricular end-diastolic diameter; MR—mitral regurgitation; RVF—right ventricular function; sPAP—systolic pulmonary artery pressure; TR—tricuspid regurgitation.

Figure 2A,B shows the association between mNEP expression on neutrophils and HFrEF severity, HFrEF etiology, and renin-angiotensin-system inhibitor therapy. Neutrophil mNEP decreased with increasing NT-proBNP tertiles (*p* = 0.001) and worsening NYHA class (*p* = 0.002). Non-ischemic etiology was characterized by higher neutrophil mNEP compared to ischemic HFrEF (5797 [IQR: 4364–7222] vs. 5108 [IQR: 3958–6433], *p* = 0.017). As shown in Table S2, individuals with ischemic HFrEF had higher NT-proBNP values (2529 pg/mL [IQR: 924–5107] vs. 1477 pg/mL [IQR: 594–3530], *p* = 0.003), worse kidney function (creatinine: 1.37 mg/dl [IQR: 1.06–1.79] vs. 1.07 mg/dl [IQR: 0.86–1.43], *p* < 0.001), and more comorbidities. Neutrophil mNEP was not different in patients receiving ARNi compared to ACEi/ARB (4834 [IQR: 3901–6913] vs. 5664 [IQR: 4507–7100], *p* = 0.101).

### 3.6. Lower Neutrophil mNEP Is Related to Adverse Cardiac Remodeling

The association of neutrophil mNEP and biventricular adverse remodeling is graphically illustrated in Figure 2C. Neutrophil mNEP decreased with worsening of left ventricular function (*p* = 0.037) and severity of mitral regurgitation (*p* = 0.005). Lower neutrophil mNEP levels were observed with worsening right ventricular function (*p* = 0.002), more severe tricuspid regurgitation (*p* = 0.009), and increasing tertiles of systolic pulmonary artery pressures (*p* = 0.012).

### 3.7. Low Neutrophil mNEP Is a Risk Factor for Future CV Hospitalization and Mortality

**Figure 3:**
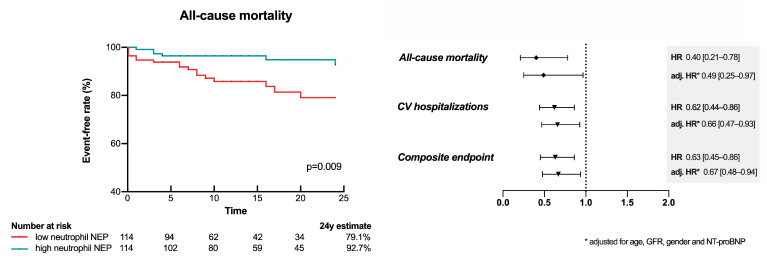
Neutrophil membrane-associated neprilysin (mNEP) expression and outcome. Unadjusted and adjusted effects of neutrophil mNEP expression on all-cause mortality, total cardiovascular (CV) hospitalizations, and the composite endpoint of both in HFrEF patients. Hazard ratios (HR) refer to a 1-interquartile range (IQR) increase in continuous variables. Hazard ratios (HR) are adjusted (adj.) for influencing variables, i.e., age, glomerular filtration rate (GFR), sex, and N-terminal pro-brain natriuretic peptide (NT-proBNP). Kaplan–Meier analysis for the primary outcome all-cause mortality with low and high neutrophil mNEP expression with the median MFI as the cut-off value. Comparison was calculated by the log-rank test.

Over a median follow-up time of 14 months (IQR: 7–24), 23 patients (10%) met the endpoint of all-cause death. Total CV hospitalization occurred in 89 (39%) and the composite endpoint of all-cause mortality or total CV hospitalizations in 95 (42%) cases. In the univariate model higher neutrophil mNEP expression was associated with reduced risk for all-cause mortality (HR per 1-IQR increase: 0.40 [0.21–0.78], *p* = 0.007), total CV hospitalization (HR per 1-IQR increase: 0.62 [0.44–0.86], *p* = 0.004), and the composite endpoint of both (HR per 1-IQR increase: 0.63 [0.45–0.86], *p* = 0.004). Neutrophil mNEP expression remained statistically significant after adjustment for a comprehensive clinical confounder model including age, kidney function, sex, and NT-proBNP to predict all-cause mortality (HR per 1-IQR increase: 0.49 [0.25–0.97], *p* = 0.039), total CV hospitalization (HR per 1-IQR increase: 0.66 [0.47–0.93], *p* = 0.019), and the composite endpoint of both (HR per 1-IQR increase: 0.67 [0.48–0.94], *p* = 0.019). The association of neutrophil mNEP and outcome is graphically illustrated in Figure 3. Figure S1 shows Kaplan–Meier curves for the secondary endpoints.

## 4. Discussion

**Figure 4:**
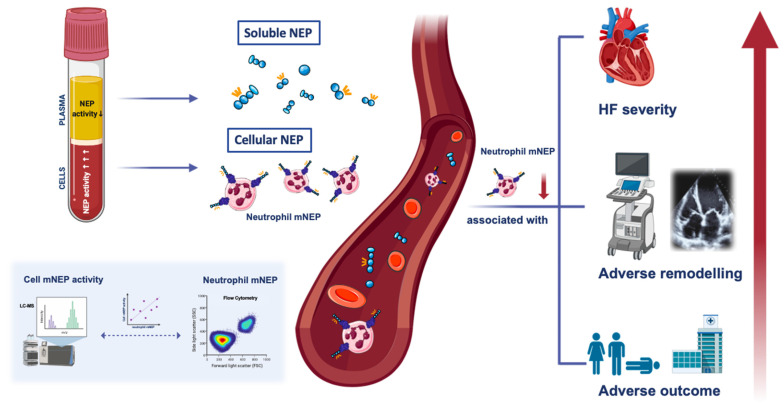
Central illustration. Neprilysin (NEP) can be released in the circulation by ectodomain shedding resulting in a soluble form of NEP. Besides the non-membrane associated form of NEP, NEP can be detected on neutrophils (cellular transmembrane NEP [mNEP]) by flow cytometry. Low neutrophil mNEP is significantly associated with progressing heart failure severity, reflected by New York Heart Association classification and N-terminal pro-brain natriuretic peptide, adverse remodeling, and poor prognosis. NEP activity of the circulating cellular compartment determined by liquid chromatography-tandem mass spectrometry (LC-MS) is markedly increased compared with plasma and correlates with neutrophil mNEP expression.

This report represents the first comprehensive description of the characteristics and prognostic role of neutrophil mNEP expression in patients with stable HFrEF including patients receiving ARNi. The present study demonstrates that (i) neutrophil mNEP rather than sNEP is the principal contributor to circulating NEP activity, (ii) HF is characterized by low neutrophil mNEP expression, and (iii) low neutrophil mNEP expression is related to a more severe HFrEF disease state reflected by neurohumoral activation, functional class, cardiac remodeling, and outcome (central illustration, Figure 4).

### 4.1. Characteristics and Significance of Neutrophil mNEP within the Circulation

In the present investigation, neutrophil mNEP was found to be a rather robust marker in stable HFrEF patients. The biologic activity of neutrophil NEP has been demonstrated earlier by experimental data showing that phosphoramidon, a potent inhibitor of NEP, almost completely blocked NEP-mediated neuropeptide degradation in neutrophil lysates [27]. A previous study investigating NEP activity in plasma and blood cellular fractions in 27 healthy volunteers reported comparable NEP activity levels in both compartments [28]. However, despite these similar values, the authors found that the degradation of atrial natriuretic peptide was exclusively attributable to NEP expressed on neutrophils. It is noteworthy that this is the first study to determine NEP activity in both plasma and blood cellular fractions in patients with HFrEF. According to our data neutrophil mNEP activity rather than plasma sNEP activity is the major contributor to circulating NEP activity. This observation may explain the so far inconclusive data on circulating sNEP measurements when investigated as a biomarker in HF.

### 4.2. Neutrophil mNEP in HFrEF: A Marker for Disease Severity

According to our data, neutrophil mNEP expression is markedly reduced in individuals with HF as compared to healthy controls with no differences observed between HFpEF and HFrEF. HFrEF patients with ischemic etiology had significantly lower neutrophil mNEP levels than patients with dilated etiology and represented a more diseased patient population. Moreover, low mNEP neutrophil expression was associated with a more severe clinical HF state, adverse remodeling, and worse prognosis. Previous studies investigating the relation of sNEP activity and circulatory natriuretic peptides in HF patients but also in non-HF cohorts reported an inverse association between sNEP activity and circulatory BNP levels [17,29]. Similarly, in the current report high levels of NT-proBNP were associated with low neutrophil mNEP expression. In contrast to sNEP activity, as of yet, there is no evidence demonstrating an association of sNEP concentration with markers of HF severity in individuals with HFrEF [10,11,15,16]. In a translational model of ischemic cardiomyopathy, NEP was downregulated with lower activity within most investigated organs and no correlation could be observed between tissue and sNEP status [30].

Several recent reports proposed a link between NEP status and cardiac remodeling by showing reverse effects of NEP inhibition [31,32]. The PROVE-HF study reported that HFrEF patients treated with ARNi show favorable morphologic and functional changes [31]. Another report demonstrated that treatment with ARNi results in significantly reduced secondary functional mitral regurgitation among patients with chronic HF [32]. In a pressure overload rat model, treatment with ARNi prevented the development of maladaptive right ventricular structural and functional changes [33]. In the present report, we observed an inverse association between neutrophil mNEP and echocardiographic indices of cardiac remodeling with a more pronounced association with markers of right heart failure. Consistent with our observation, a study conducted in patients with hypertrophic cardiomyopathy reported an inverse relationship of sNEP with left and right ventricular function [34]. To our knowledge, no prior studies have examined the association of echocardiographic indices and circulatory NEP measures in chronic HFrEF patients.

The prognostic role of circulatory NEP measures has been discussed controversially in the past. Most studies could not confirm the association of sNEP concentrations with outcome in different HF collectives but also non-HF cohorts [11,12,13,14,17]. Yet, the prognostic impact of sNEP activity has rarely been investigated. One report conducted in patients with chronic kidney disease described an inverse relationship between sNEP activity and HF hospitalizations [17], while another study including chronic HFrEF could not find an association with outcome [11]. Data from this study suggest that higher neutrophil mNEP levels are associated with better overall survival and less frequent CV hospitalizations in HFrEF.

### 4.3. Possible Links between Neutrophil mNEP and Heart Failure: Vasoactivity and Inflammation

NEP is critical for the processing and catabolism of numerous vasoactive peptides involved in natriuresis and diuresis, e.g., natriuretic peptides, adrenomedullin, angiotensin II, and endothelin [4,5,6]. It has been hypothesized that the mechanism of action of NEPi in HFrEF is based on a beneficial net effect on vasoactive peptide homeostasis. As the actions of NEP are assumed to be primarily tissue based, involvement of circulating neutrophil mNEP in systemic vasoregulation may equally be assumed. Moreover, previous data indicate that neutrophil mNEP plays a role in chemotaxis and neutrophil responsiveness to inflammatory stimuli by modulating proinflammatory peptides such as formyl-Met-Leu-Phe, substance P, and enkephalins [19]. Therefore, the expression of NEP on neutrophils may contribute to both the regulation of the vascular tone and local inflammatory responses.

Chronic subclinical inflammation plays a critical role in the development and progression of HF [35,36,37]. Target disruption studies demonstrated the crucial involvement of NEP in the regulation of the inflammatory reaction as NEP deficient mice were found to be 10-fold more susceptible to endotoxin and died 100-fold more rapidly from septic shock [20]. Moreover, neutrophil NEP status has been shown to be indicative for the maturation status of neutrophils, while low levels of mNEP characterize immature neutrophils [38,39,40,41]. This subpopulation shows an aberrant function and is found in cancer, infections, systemic inflammation and also pregnancy [21,42,43,44]. It has been previously proposed that the presence of neutrophil precursors in circulation may result from a shift from steady-state hematopoiesis to emergency granulopoiesis in order to respond to an increased demand of neutrophils during stress or systemic inflammation [45]. In a recent study conducted in patients with ST-elevation myocardial infarction, immature neutrophils were found to independently predict poor clinical outcome [46]. The observed association of low neutrophil mNEP expression with severe HFrEF in this study might reflect a significantly compromised immunological regulation in advanced disease state.

### 4.4. Implications for Clinical Practice

These findings seem intriguing given the fact that the inhibition, not the activation of NEP is associated with clinical benefits [1]. On the other hand, the effect of ARNi seems to be more pronounced in less advanced HF stages reflected by a lower number needed to treat [47], which might be due to higher NEP levels in this population. In both experimental and human HF, previous studies supported the hypothesis that an excessive production of natriuretic peptides results in reduced response to natriuretic peptides assumedly due to downregulation and inactivation of receptors [48,49]. Several lines of evidence suggest that high circulating BNP concentrations might have an auto-inhibitory effect on NEP [29,50]. Lower NEP expression and the accumulation of natriuretic peptides would both result in lower baseline NEP activity and may render NEP less attractive as a pharmacologic target in severe HF. In this context, an early initiation of treatment with ARNi before HF progresses to an NEPi-unresponsive disease stage may be of clinical relevance.

## 5. Limitations

As clinical characteristics were only assessed at study inclusion, data on the effects of clinical changes on neutrophil mNEP expression cannot be provided. As such, future studies with multiple assessment times are required to further clarify the prognostic implications of neutrophil mNEP levels.

## 6. Conclusions

This study provides first data on the role of neutrophil mNEP within the context of HF. The measurement of the integer form of mNEP uniquely extends previous studies on circulating NEP measures aiming at finding a relationship between individual NEP status and HF disease state. The data indicate that low neutrophil mNEP expression in HFrEF patients is related to progressing HF severity, adverse remodeling and worse prognosis. The results underscore the importance of early initiation of ARNi in less severe HF disease, where NEPi-response could be more pronounced.

## Figures and Tables

**Table 1:** Baseline characteristics for the HFrEF cohort. Continuous variables are given as medians and interquartile ranges (IQR), counts are given as numbers and percentages.

Baseline Characteristics	Total Study Population (n = 228)
Age, median years (IQR)	64 (55 to 72)
Male gender, n (%)	169 (74)
BMI, kg/m^2^ (IQR)	28 (24 to 32)
Systolic blood pressure, mmHg (IQR)	122 (110 to 140)
Diastolic blood pressure, mmHg (IQR)	75 (70 to 85)
Heart rate, min^−1^ (IQR)	69 (62 to 80)
NYHA functional class	
NYHA I, n (%)	36 (16)
NYHA II, n (%)	97 (43)
NYHA III, n (%)	91 (40)
NYHA IV, n (%)	4 (2)
**Comorbidities**	
Ischemic etiology of HF, n (%)	101 (44)
Non-ischemic etiology of HF, n (%)	127 (56)
Hypertension, n (%)	130 (57)
Type II diabetes mellitus, n (%)	76 (33)
Atrial fibrillation, n (%)	81 (36)
**Laboratory parameters**	
Hemoglobin, g/dl (IQR)	13.5 (12.2 to 14.5)
WBC, G/l (IQR)	7.14 (6.04 to 8.75)
Neutrophil count, G/l (IQR)	4.5 (3.7 to 5.7)
Serum creatinine, mg/dl (IQR)	1.19 (0.91 to 1.66)
Blood urea nitrogen, mg/dl (IQR)	22.8 (16.7 to 33.7)
Total cholesterol, mg/dl (IQR)	168 (134 to 192)
C-reactive protein, mg/dl (IQR)	0.29 (0.14 to 0.85)
Total bilirubin, mg/dl (IQR)	0.60 (0.43 to 8.40)
BChE, kU/I (IQR)	7.07 (5.52 to 8.81)
NT-proBNP, pg/mL (IQR)	1819 (746 to 4264)
**Medication**	
Beta-blocker, n (%)	217 (95)
Diuretics, n (%)	102 (45)
Mineralocorticoidantagonist, n (%)	180 (79)
I_f_ Inhibitor (%)	21 (9)
ACE-I/ARB/ARNI, n (%) Dose equivalent, ≥50%	101/50/62 (44/22/27) (92/86/79)
**Echocardiographic characteristics**	
Left ventricular end-diastolic diameter, mm	58 (52 to 65)
Left ventricular function (≥ moderately reduced), n (%)	195 (86)
Mitral regurgitation (≥ moderate), n (%)	124 (54)
Right ventricular end-diastolic diameters, mm	37 (32 to 42)
Right ventricular function (≥ moderately reduced), n (%)	82 (36)
Tricuspid regurgitation (≥ moderate), n (%)	106 (46)
Systolic pulmonary artery pressure, mmHg	48 (37 to 59)

IQR—interquartile range; BMI—body mass index; NYHA—New York Heart Association; HF—heart failure; WBC—white blood count; BChE—butyrylcholinesterase; NT—proBNP–N-terminal pro-B-type-natriuretic peptide; ACE-I—angiotensin converting enzyme inhibitor; ARB—angiotensin II receptor blocker; ARNI—angiotensin receptor-neprilysin inhibitor.

## Data Availability

The data supporting the findings of this study can be made available from the corresponding author upon reasonable request.

## Supplementary Materials

The following are available online at https://www.mdpi.com/article/10.3390/cells10112922/s1, Figure S1: Kaplan–Meier curves for total cardiovascular (CV) hospitalizations (A) and the composite endpoint of all-cause mortality and total CV hospitalizations (B) in HFrEF patients. Table S1: Baseline characteristics presented for the HFrEF, HFpEF, and control group. Table S2. Baseline characteristics presented for the HFrEF group stratified by heart failure etiology.
