# Supplementary material for: Relevance of Neutrophil Neprilysin in Heart Failure
Source: Cells. 2021 Oct 28;10(11):2922. doi: 10.3390/cells10112922 (PMC8616455; doi:10.3390/cells10112922)
Supplement: Supplementary file 1 [file cells-10-02922-s001.zip › cells-1410320-supplementary.pdf]

SUPPLEMENTAL MATERIAL

Supplemental Tables

Table S1. Baseline characteristics presented for the HFrEF, HFpEF and control group.

Baseline characteristics	HFrEF (n=228)	HFpEF (n=30)	Controls (n=43)
Age, median years (IQR)	64 (55 to 72)	76 (71 to 79)	35 (27 to 51)
Male gender, n (%)	169 (74)	8 (27)	19 (44)
BMI, kg/m^2 (IQR)	28 (24 to 32)	27 (26 to 30)	-
Systolic blood pressure, mmHg (IQR)	122 (110 to 140)	130 (119 to 146)	-
Diastolic blood pressure, mmHg (IQR)	75 (70 to 85)	80 (70 to 86)	-
Heart rate, min^{-1} (IQR)	69 (62 to 80)	72 (63 to 79)	-
NYHA I, n (%)	36 (16)	4 (13)	0 (0)
NYHA II, n (%)	97 (43)	12 (40)	0 (0)
NYHA III, n (%)	91 (40)	13 (43)	0 (0)
NYHA IV, n (%)	4 (2)	1 (3)	0 (0)
**Comorbidities**			
Non-ischemic etiology of HF, n (%)	127 (56)	-	0 (0)
Hypertension, n (%)	130 (57)	23 (77)	0 (0)
Type II diabetes mellitus, n (%)	76 (33)	5 (17)	0 (0)
Atrial fibrillation, n (%)	81 (36)	16 (53)	0 (0)
**Laboratory parameters**			
Hemoglobin, g/dl (IQR)	13.5 (12.2 to 14.5)	12.2 (11.1 to 13.7)	13.8 (13.0 to 14.7)
WBC, G/l (IQR)	7.14 (6.04 to 8.75)	6.60 (5.31 to 8.23)	5.68 (5.02 to 7.24)
Serum creatinine, mg/dl (IQR)	1.19 (0.91 to 1.66)	1.20 (0.92 to 1.39)	0.82 (0.67 to 0.93)
Blood urea nitrogen, mg/dl (IQR)	22.8 (16.7 to 33.7)	23.4 (17.5 to 30.0)	11.2 (9.0 to 14.3)
Total cholesterol, mg/dl (IQR)	168 (134 to 192)	166 (133 to 188)	175 (154 to 204)
C-reactive protein, mg/dl (IQR)	0.29 (0.14 to 0.85)	0.31 (1.14 to 0.58)	0.07 (0.03 to 0.15)
Total bilirubin, mg/dl (IQR)	0.60 (0.43 to 8.40)	0.48 (0.32 to 0.95)	0.50 (0.39 to 0.69)
BChE, U/I (IQR)	7.07 (5.52 to 8.81)	6.95 (5.36 to 8.06)	7.45 (6.88 to 8.45)

NT-proBNP, pg/ml (IQR)	1819 (746 to 4264)	969 (554 to 1838)	41 (24 to 75)
**Medication**			
Beta-blocker, n (%)	217 (95)	25 (83)	0 (0)
Diuretics, n (%)	102 (45)	13 (43)	0 (0)
Mineralocorticoidantagonist, n (%)	180 (79)	12 (40)	0 (0)
I_f Inhibitor (%)	21 (9)	1 (3)	0 (0)
ACE-I/ARB/ARNI, n (%)	101/50/62 (44/22/27)	4/11/0 (13/37/0)	0 (0)
**Echocardiographic characteristics**			
Left ventricular end-diastolic diameter, mm	58 (52 to 65)	45 (41 to 48)	-
Left ventricular function (≥ moderately reduced), n (%)	195 (86)	0	-
Mitral regurgitation (≥ moderate), n (%)	124 (54)	3 (10)	-
Right ventricular end-diastolic diameters, mm	37 (32 to 42)	35 (32 to 40)	-
Right ventricular function (≥ moderately reduced), n (%)	82 (36)	0	-
Tricuspid regurgitation (≥ moderate), n (%)	106 (46)	15 (50)	-
Systolic pulmonary artery pressure, mmHg	48 (37 to 59)	51 (41 to 64)	-

IQR – interquartile range, BMI – body mass index, NYHA – New York Heart Association, HF – heart failure, WBC – white blood count, BChE – butyrylcholinesterase, NT – proBNP – N-terminal pro-B-type-natriuretic peptide, ACE-I – angiotensin converting enzyme inhibitor, ARB – angiotensin II receptor blocker, ARNI – angiotensin receptor-neprilysin inhibitior

Table S2. Baseline characteristics presented for the HFrEF group stratified by heart failure etiology.

Baseline characteristics	non-ischemic CMP (n=127)	ischemic CMP (n=101)	p-value
Age, median years (IQR)	60 (47 to 69)	69 (61 to 75)	<0.001
Male gender, n (%)	84 (66)	85 (84)	0.002
Systolic blood pressure, mmHg (IQR)	130 (110 to 140)	120 (105 to 135)	0.105
Diastolic blood pressure, mmHg (IQR)	78 (70 to 90)	74 (70 to 80)	0.068
BMI, kg/m^2 (IQR)	27 (24 to 32)	28 (24 to 32)	0.296
NYHA functional class			<0.001
NYHA I, n (%)	29 (23)	7 (7)	
NYHA II, n (%)	58 (46)	39 (39)	
NYHA III, n (%)	39 (31)	52 (52)	

NYHA IV, n (%)	1 (1)	3 (3)	
**Comorbidities**			
Hypertension, n (%)	54 (43)	76 (75)	<0.001
Type II diabetes mellitus, n (%)	25 (20)	51 (50)	<0.001
Atrial fibrillation, n (%)	42 (33)	39 (39)	0.355
**Laboratory parameters**			
Hemoglobin, g/dl (IQR)	13.7 (12.6 to 14.9)	13.3 (11.6 to 14.2)	0.007
WBC, G/l (IQR)	7.27 (6.15 to 9.08)	7.12 (5.84 to 8.61)	0.331
Serum creatinine, mg/dl (IQR)	1.07 (0.86 to 1.43)	1.37 (1.06 to 1.79)	<0.001
Total cholesterol, mg/dl (IQR)	173 (147 to 199)	158 (125 to 183)	0.003
C-reactive protein, mg/dl (IQR)	0.27 (0.14 to 0.66)	0.30 (1.14 to 1.05)	0.253
BChE, kU/I (IQR)	7.42 (5.57 to 8.78)	6.78 (5.27 to 8.91)	0.173
NT-proBNP, pg/ml (IQR)	1477 (594 to 3530)	2529 (924 to 5107)	0.003

CMP - cardiomyopathy, IQR – interquartile range, BMI – body mass index, NYHA – New York Heart Association, WBC – white blood count, BChE – butyrylcholinesterase, NT – proBNP – N-terminal pro-B-type-natriuretic peptide.

Supplemental Figures

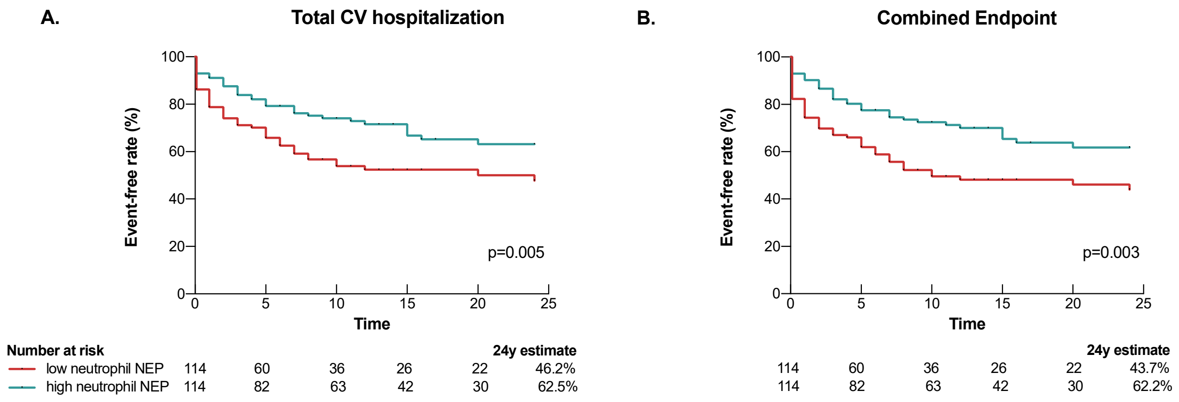

Figure S1. Kaplan-Meier curves for total cardiovascular (CV) hospitalizations (A) and the composite endpoint of all-cause mortality and total CV hospitalizations (B) in HFrEF patients. Low and high neutrophil NEP expression with the median MFI as the cut-off value. Comparison was calculated by the log-rank test.
